# Regulation of the oncogenic phenotype by the nuclear body protein ZC3H8

**DOI:** 10.1186/s12885-018-4674-1

**Published:** 2018-07-24

**Authors:** John A. Schmidt, Keith G. Danielson, Emily R. Duffner, Sara G. Radecki, Gerard T. Walker, Amber Shelton, Tianjiao Wang, Janice E. Knepper

**Affiliations:** Department of Biology, Mendel Science Center, Villanova University, 800 East Lancaster Avenue, Villanova, PA 19085 USA

**Keywords:** PML body, Cajal body, LEC (little elongation complex), Cell migration, Cell invasion, Zinc finger protein, 3-D cell growth

## Abstract

**Background:**

The *Zc3h8* gene encodes a protein with three zinc finger motifs in the C-terminal region. The protein has been identified as a component of the Little Elongation Complex, involved in transcription of small nuclear RNAs. *ZC3H8* is overexpressed in a number of human and mouse breast cancer cell lines, and elevated mRNA levels are associated with a poorer prognosis for women with breast cancer.

**Methods:**

We used RNA silencing to decrease levels of expression in mouse mammary tumor cells and overexpression of ZC3H8 in cells derived from the normal mouse mammary gland. We measured characteristics of cell behavior in vitro, including proliferation, migration, invasion, growth in soft agar, and spheroid growth. We assessed the ability of these cells to form tumors in syngeneic BALB/c mice. ZC3H8 protein was visualized in cells using confocal microscopy.

**Results:**

Tumor cells with lower ZC3H8 expression exhibited decreased proliferation rates, slower migration, reduced ability to invade through a basement membrane, and decreased anchorage independent growth in vitro. Cells with lower ZC3H8 levels formed fewer and smaller tumors in animals. Overexpression of ZC3H8 in non-tumorigenic COMMA-D cells led to an opposite effect. ZC3H8 protein localized to both PML bodies and Cajal bodies within the nucleus. ZC3H8 has a casein kinase 2 (CK2) phosphorylation site near the N-terminus, and a CK2 inhibitor caused the numerous PML bodies and ZC3H8 to coalesce to a few larger bodies. Removal of the inhibitor restored PML bodies to their original state. A mutant ZC3H8 lacking the predicted CK2 phosphorylation site showed localization and numbers of ZC3H8/PML bodies similar to wild type. In contrast, a mutant constructed with a glutamic acid in place of the phosphorylatable threonine showed dramatically increased numbers of smaller nuclear foci.

**Conclusions:**

These experiments demonstrate that *Zc3h8* expression contributes to aggressive tumor cell behavior in vitro and in vivo. Our studies show that ZC3H8 integrity is key to maintenance of PML bodies. The work provides a link between the Little Elongation Complex, PML bodies, and the cancer cell phenotype.

**Electronic supplementary material:**

The online version of this article (10.1186/s12885-018-4674-1) contains supplementary material, which is available to authorized users.

## Background

**Fig. 1:**
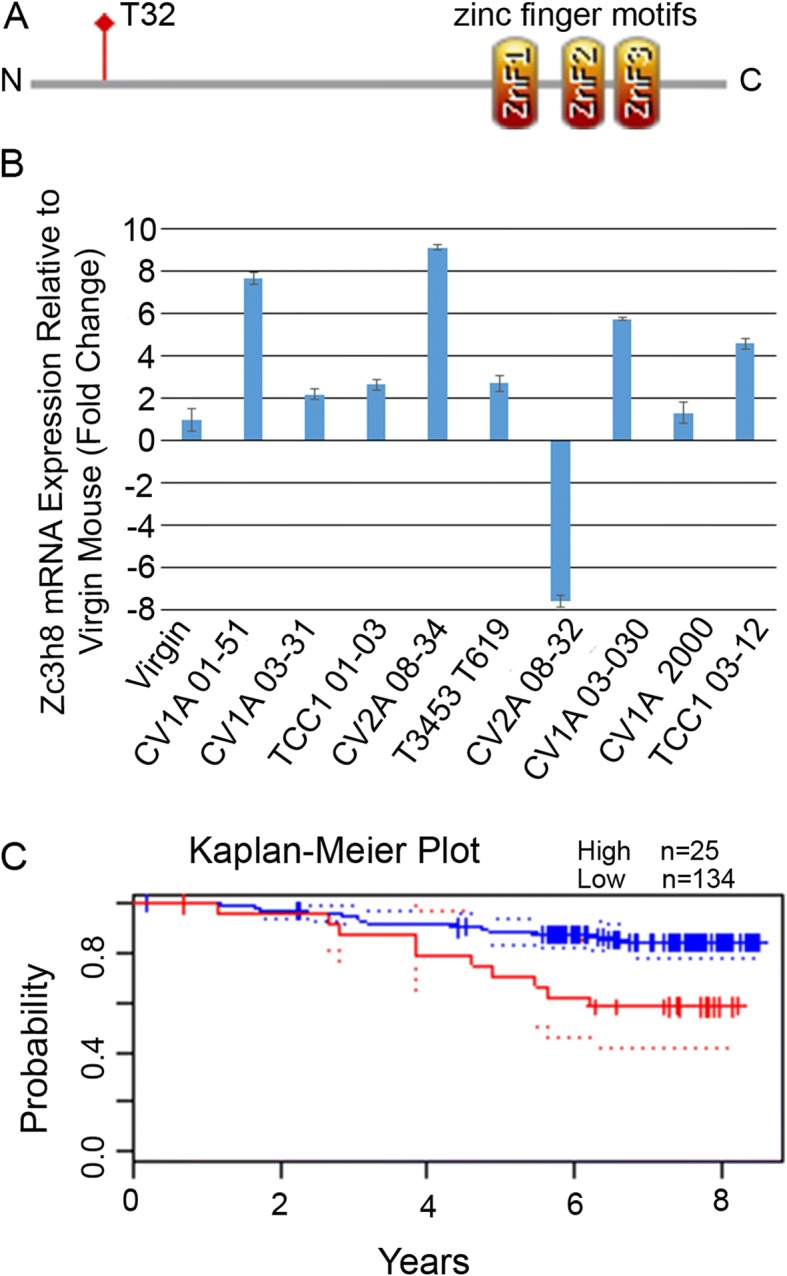
ZC3H8 predicted structure and expression in tumors. **a**) Domain map of ZC3H8 shows a putative CK2 phosphorylation site at amino acid T32 and three putative C3H1 zinc finger motifs in the c-terminus. **b**) RT-qPCR survey of tumor cell lines derived from mouse mammary tissue expressing *Zc3h8*. Experiments were performed in triplicate, error bars represent standard deviation. **c**) Kaplan-Meier plot of disease-specific survival of breast cancer with high and low expression of ZC3H8, from Prognoscan data set GSE1456-GPL97, *p* value = 0.043377, hazard ratio 2.30, 95% CI = 0.85–6.24, Cox p value = 0.100218 [24, 25]

The zinc finger protein ZC3H8 was first identified as expressed in fetal liver [1], but moderate levels can be detected in a variety of tissues, and the gene is amplified in 2–6% of solid tumors of the breast [2]. The *Zc3h8* gene encodes a protein of predicted molecular weight 34 kDa of unknown function. There are three predicted zinc fingers in the carboxy terminal domain, and a potential casein kinase 2 (CK2) phosphorylation site at threonine 32 [3] (Fig. 1a). Zinc finger proteins of this arrangement (CCCH/C3H1) are found in eukaryotes including yeasts, trypanosomes, plants, and animals and have been shown to bind RNA and be involved in post-transcriptional regulatory processes in several cases [4–11]. ZC3H8 specifically was identified in a cross-linking study of the human embryonic kidney cell proteome bound to mRNA [12]. Recent work has demonstrated that the zinc finger domains of some of the family members from disparate species are in fact, functionally interchangeable, thus suggesting a common strategy for binding RNA [13].

Hu et al. identified ZC3H8 as a component of the human little elongation complex (LEC), which functions in initiation and elongation of transcription of snRNA genes [14]. Knockdown of expression of the ICE1 subunit of the LEC led to decreased occupancy at U11 and U12 promoters, though knockdown of ZC3H8 had no effect on promoter binding [14]. Additionally, in a global analysis of snRNA expression, loss of ICE1 or ELL led to general defects in snRNA expression, while loss of ICE2 and ZC3H8 components did not affect snRNA expression [14]. Egloff et al. recently determined that ZC3H8 functions as a key component of the LEC, in combination with ICE1, ICE2, ELL, and the 7SK snRNP; when the RNA component was depleted, ZC3H8 association with the complex was abolished [15]. Takahashi et al., however, did not find evidence of ZC3H8 in complex with LEC recruited to snRNA genes by the mediator component MED26 [16], perhaps due to the existence of multiple forms of the LEC. This leaves the precise role of ZC3H8 in the LEC in question. Given the suggested role of CCCH zinc finger proteins in RNA binding, it is possible that ZC3H8 plays a role in processing or maturation of LEC transcription products rather than direct transcriptional events.

Several studies have localized the ZC3H8 protein to distinct nuclear bodies. The LEC and associated proteins, including ZC3H8, were found in Cajal bodies, colocalized with COILIN, a marker for that structure [14]. MED26, a mediator component, has also been associated with the LEC in Cajal bodies, but ZC3H8 was not linked to the complex in this study [16]. However, Fong et al. performed genome scale profiling and tandem affinity-mass spectrometric analysis of a large number of proteins localized to distinct nuclear bodies, and found instead that ZC3H8, along with many other RNA binding proteins, was found in nuclear paraspeckles [17]. These subnuclear bodies are sites of retention of edited RNAs and long non-coding RNAs involved in regulation [18, 19].

The noted amplification of ZC3H8 in human breast tumors, the potential regulatory effect of a known oncogene, CK2, and the developing understanding of post-transcriptional regulation contributing to breast cancer, provided the rationale for undertaking these experiments. The studies presented here aimed to determine if ZC3H8 expression leads to invasive tumor cell behavior, and to determine if phenotypic changes are associated with the localization of ZC3H8 in nuclear subcompartments. Our experiments support a role for ZC3H8 in tumorigenesis and suggest that localization is dependent upon the phosphorylation state of the protein.

## Methods

### Antibodies and reagents

Antibodies against ZC3H8 were from AssayBiotech (#C19576) and Lifespan BioSciences (#59503) for microscopy and blotting respectively. Antibodies against PML were from Acris (#AM20293AF-N), NONO from OneWorld (#41152), COILIN monoclonal from Abnova (#B01P), COILIN polyclonal from OneWorld (#41139), CK2 from Thermo Fisher (#PA5–28686), V5 from Millipore (#AB3792), GAPDH from GeneTex (#GTX100118) or Origene (#TA802519S), and FLAG (#2368) and β-TUBULIN (#2128) were from Cell Signaling Technologies. Secondary anti-rabbit and anti-mouse HRP-linked antibodies were from Cell Signaling Technologies and fluorophore-linked antibodies were from Thermo Fisher. All antibodies were used at concentrations recommended by the commercial suppliers. 4,5,6,7-Tetrabromobenzotriazole (TBB) was purchased from Tocris. Quinalizarin was obtained from Sigma Aldrich.

### Cloning and vectors

Coding sequences for mouse *Zc3h8* were amplified from cDNA from CV1A 03–31 mouse mammary carcinoma cells using primers For: 5’-CCCTGTCTGAGTTATGGATTTTG-3′, and Rev.: 5’-TTTACATGACTTCTTGTCAGTATC-3′. Human ZC3H8 was similarly obtained by RT-PCR of RNA from MCF7 cells using primers For: 5’-GACCTGTCTGGGTCATGGATTTG-3′ and Rev.: 5’-TTTACATGACTTCTTTTCAGTATC-3′. Amplified sequences were subsequently inserted into the TA cloning site of pcDNA 3.1 V5 His and pEF6 V5 (Thermo Fisher). Knockdown sequences shRNA A: 5’-GATCCCGGGACCGTGAACAAATTCTTCAAGAGAGAATTTGTTCACGGTCCCGTTA-3′ and its inverse complement 5’-AGCTTAACGGGACCGTGAACAAATTCTCTCTTGAAGAATTTGTTCACGGTCCCGG-3′ targeting bases 241–259 in the *Zc3h8* sequence and shRNA C: 5’-GATCCCGCAAAGGGAAGCAAGTTTTTCAAGAGAAAACTTGCTCCCTTTGCGTA-3′ and the inverse complement 5’-AGCTTAACGCAAAGGGAAGCAAGTTTTCTCTTGAAAAACTTGCTTCCC TTTGCGG-3′) targeting bases 709–727 were cloned into pSilencer 4.1 (Ambion/ThermoFisher) according to the manufacturer’s directions. Negative control plasmids provided by Ambion contain sequences not found in the human, mouse, or rat genome databases. For site-directed mutagenesis of *Zc3h8*, the Q5 kit (New England Biolabs) was used according to manufacturer directions. Two primers were used to create T32A and T32E mutations in mouse *Zc3h8*: 5′- GATGAAATAGATGGTGCTGAAGTTGAAGAAACACAGACAG-3′, 5′ -GATGAAATAGATGGTGAGGAAGTTGAAGAAACACAGACAG- 3′. Each PCR reaction used the same reverse primer 5’-AATTCTTTCCTCAGGGTCGGCGGCC-3′ per the supplier’s instructions. All cloning and mutagenesis experiments were verified by DNA sequencing through Genewiz.

### Cell culture

Mouse mammary tumor cell lines were derived from independently arising tumors in BALB/cV mice carrying the MMTV cV [20, 21]; or BALB/c mice carrying a chemically induced mammary tumor expressing a variant MMTV strain of unknown origin [22]. Tumors were excised, minced and grown in DMEM supplemented with 50% fetal bovine serum (FBS), decreased in 10% increments at each passage to a final 10% FBS. Mouse mammary cV1A 03–31, cV1A 01–51, or COMMA-D cells [23] were grown in DMEM with 10% FBS at 37 °C with 100% humidity and 5% CO_2_. For treatments with TBB, cells were washed three times with DMEM without serum and incubated with either DMSO or 10 μM TBB in serum-free DMEM for 2 h in the incubator. To remove the TBB, cells were subsequently washed with DMEM full medium three times and allowed to recover for 2 h under normal conditions. Experiments using the alternative CK2 inhibitor quinalizarin were performed using the same conditions except that quinalizarin was used at 30 μ*M* because of its reduced cell permeability. Cell transfections were done using Lipofectamine (Thermo Fisher) reagent according to manufacturer’s instructions. For stable cell line selection for RNA silencing, single colonies were selected using 500 μg/ml Geneticin treatments for 2 weeks followed by screening for expression using RT-qPCR or western blot analysis. Stable COMMA-D derivatives stably overexpressing *Zc3h8* were selected using 3 μg/ml blasticidin (Thermo Fisher).

### RNA extraction and RT-qPCR

RNA was extracted from mitotically active cell cultures using Tri-Reagent (Sigma/Aldrich) according to the manufacturer’s protocol. RNA was converted to cDNA using a VILO kit (Thermo Fisher) and the product diluted in RNase-free water prior to RT-qPCR using Power-UP SYBR Green qPCR master mix (Thermo Fisher). PCR reactions were performed in triplicate, and experiments were repeated. Primers used were: *Zc3h8* For: 5′- CCGCCGACCCTGAGGAAAGAATTG-3′, Rev.: 5’-GGAAGTAATGAGGGTTGAGCTGCGT-3′; *Gata-3* For: 5’-AGGGACATCCTGCGCGAACTGT-3′, Rev.: 5’-CATCTTCCGGTTTCGGGTCTGG-3′; *Act1* For: 5’-GACGGCCAGGTCATCACTATTG-3′, Rev.: 5’-AGGAAGGCTGGAAAAGAGCC-3′, *Gapdh* For: 5’-GACAACTTTGGCATTGTGG-3′, Rev.: 5’-ATGCAGGGATGATGTTCTG-3′.

### Fluorescence microscopy

Cells were grown on No. 1.5 glass coverslips for 24 h with complete medium followed by any indicated treatments. Fixatives, permeabilization solutions, and antibodies were prepared in phosphate buffered saline (1 X PBS). Coverslips were fixed for 10 min in 4% formaldehyde. Coverslips were washed in PBS three times for 5 min each. Cells on the coverslips were then permeabilized in a 0.1% Triton X-100 for 10 min. Cells were then incubated with primary antibodies for 1 h at room temperature and then washed three times in PBS for 5 min each and incubated with secondary antibodies for 1 h at room temperature. Antibody dilutions were: anti CK2 1:100 (Thermo Scientific), anti V5 1:100 (Millipore), anti ZC3H8 1:100 (Assay Biotech), anti COILIN 1:100 (monoclonal, Abnova) or 1:100 (Thermo, polyclonal), anti PML 1:2000 (Acris), anti DAXX 1:25 (Cell Signaling), anti SMN1 1:100 (Cell Signaling), and anti NARG2/ICE2 1:100 (Bioss). Cells were washed three more times in PBS for 5 min each then mounted on slides with Vectashield +DAPI and sealed with nail polish. Images were captured and processed using a Leica SP8 confocal microscope system with a Leica 63X oil immersion objective.

### Migration and invasion assays

Wound healing assays were done using silicone inserts from Ibidi following the manufacturer’s directions. Images were captured using a 10X objective on an Olympus IX70 microscope. Statistical analysis was determined by ANOVA. Corning Biocoat Matrigel Invasion Chambers with 8.0 μm pore inserts were purchased and used according to the manufacturer’s directions. Briefly, a chemoattractant of 5% FBS in DMEM was used in the lower chamber and 2.5 × 10^4^ cells in 500 μl DMEM were added to the upper chamber and incubated for 22 h. Non-migrating cells were scraped off and migrated cells were fixed with 4% paraformaldehyde and stained with crystal violet. Cells were imaged and counted using a Leica ICC50 HD Microscope. *P* values were determined by Tukey post hoc. Experiments were performed in triplicate and each was repeated. Error bars represent standard deviation.

### Assays for 3-D growth

For soft agar assays, a 1 mL layer of 1.5% agarose in PBS was solidified in a 35 mm tissue culture dish and equilibrated with DMEM with 10% FBS. A subsequent layer of 2 mL of 0.5% low melting point agarose with suspended cells in DMEM and 10% FBS was layered on top and covered with 0.5 ml of liquid medium. A total of 5 × 10^4^ cells/well in each of three wells was seeded and incubated for 2 weeks. Fresh media was exchanged every 3 days. Colonies were imaged and quantified using an Olympus IX70 inverted microscope. P values were determined by Student’s t-test. For in vitro 3D spheroid growth assays, 5 × 10^3^ cells per well were added to eight wells per experiment and imaged every 24 h and *p* values were determined by ANOVA. Corning round well-bottom 96-well dishes with ultra-low attachment surface coating were used (cat. 4520). Error bars represent standard deviation. All 3-D growth assays were repeated three times.

### Cell proliferation assay

Cell proliferation assays used the CellTiter-Glo 2.0 Luminescent Cell Viability kit (Promega). Briefly, 5 × 10^3^ cells (100 μL at 5 × 10^4^ cells/mL) were added to an opaque 96-well dish and incubated for 24 h. 100 μL of reagent was added to the wells and incubated for 10 min at room temperature and measured for luciferase activity using a CLARIOstar Plate Reader (BMG Labtech). Cell-free wells were used as blanks and each read included eight replicate wells per cell line per experimental condition. Measurements were taken every 24 h for 5–7 days. Each experiment was repeated three times. Statistical significance was assessed by ANOVA.

### Protein extraction and western blot analysis

Cell lysates were extracted using RIPA Buffer (Thermo Fisher) with protease and phosphatase inhibitors (Phosphatase Inhibitor Cocktail II and Protease Inhibitor Cocktail III for mammalian cells, Research Products International). Equal amounts of protein (30 μg) were separated by SDS PAGE (10% gel) and transferred to PVDF membrane for blotting. Protein detection was done with primary and secondary antibodies and enhanced chemiluminescence reagent (Thermo Fisher). Densitometry to determine relative protein levels used ImageJ (NIH).

### Tumor growth

All experiments utilizing animals were performed according to protocols approved by Villanova University’s Animal Care and Use Committee. Subcutaneous injections of 1 × 10^6^ cells were injected into 6–8 wk. old female BALB/c mice and monitored for 8 weeks or until tumors reached 1 cm diameter. Mice were euthanized using CO_2_ and tumors were dissected and weighed. Error bars represent standard deviation, and *p* values were determined by Student’s t- test.

### Prognoscan

Kaplan-Meier plots were generated using the Prognoscan website, Prognoscan.org, data set GSE1456-GPL97, *p* value = 0.043377 [24]. The data set used is from the Breast Cancer Stockholm 1994–1996 cohort [25]. Other breast cancer data sets showed similar trends.

Supplementary Information accompanies this paper.

## Results

### Zc3h8 expression in mouse mammary carcinoma cells

We identified a potential role for Zinc Finger CCCH-Type containing 8 (*Zc3h8)* in tumorigenesis using PCR to locate integration sites of the mouse mammary tumor virus (MMTV) in tumors of BALB/cV mice. The expected result of MMTV integration would be overexpression of the target site locus [26]. We derived a number of cell lines from mouse mammary tumors and screened these for *Zc3h8* expression compared to virgin mammary gland tissue or normal mouse mammary epithelial cells. We found that 7 of 9 tested mouse mammary carcinoma cell lines have 2 fold or greater elevated expression of *Zc3h8* by examining mRNA levels using RT-qPCR (Fig. 1b). One cell line expressed quite low levels of *Zc3h8*; the cV2A 08–32 line grows slowly in culture. *Zc3h8* may therefore have a role in tumorigenesis. Online datasets also indicated a relationship between carcinogenesis, disease-free survival and *ZC3H8* expression. Fig. 1c shows a sample Kaplan-Meier Plot of 159 individuals with breast cancer generated using Prognoscan [24]. High expression of *ZC3H8* decreases the probability of disease-specific survival, a trend seen in many datasets for breast and other cancers. The human protein atlas (https://www.proteinatlas.org/) designates *ZC3H8* as an unfavorable prognostic marker for endometrial and renal cancers [27].

### Reduced expression of Zc3h8 in cells affects cell behavior

**Fig. 2:**
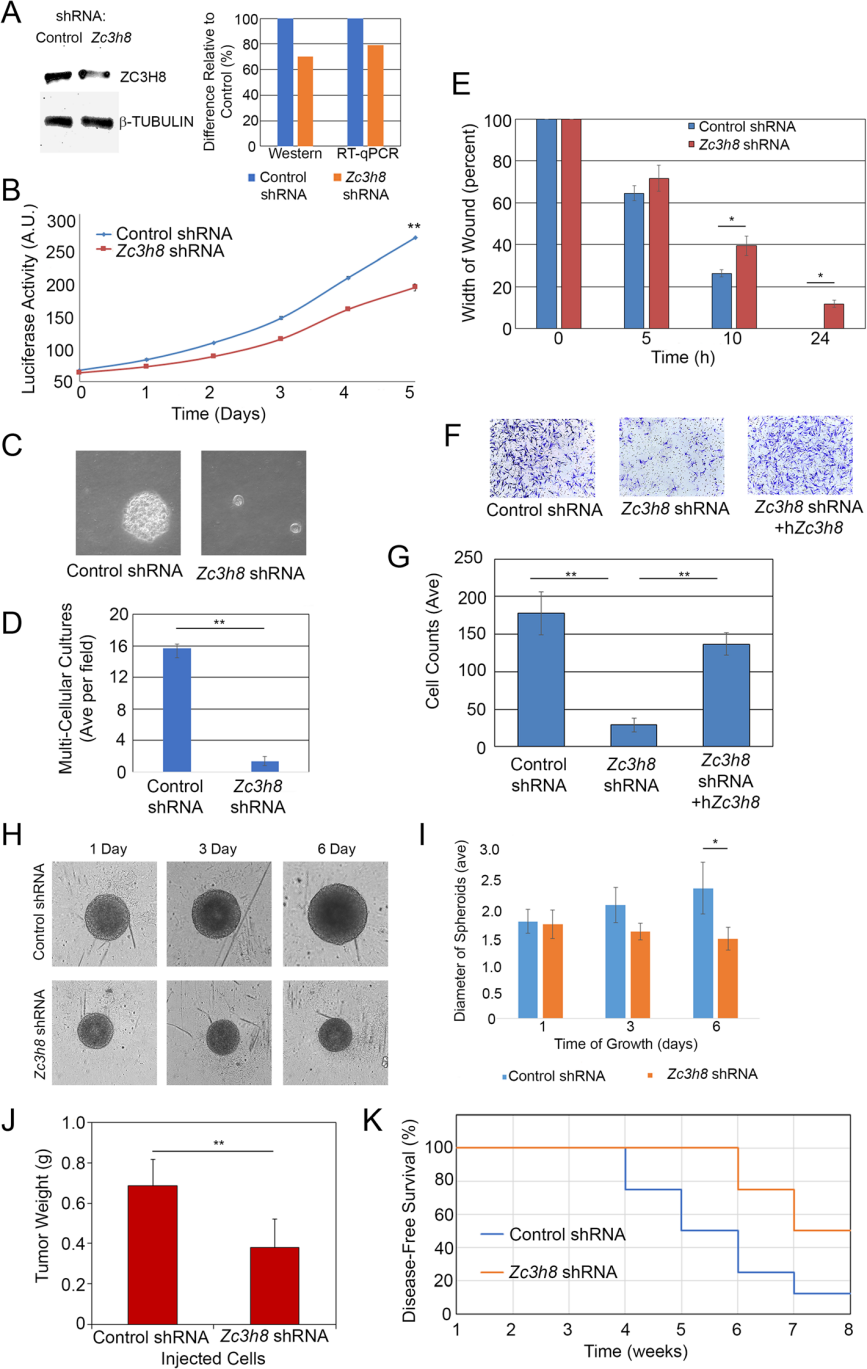
Reduced expression of *Zc3h8* leads to reduced invasive growth in vitro and in vivo. **a**) cV1A 03–31 mouse mammary carcinoma cells stably expressing shRNA targeting *Zc3h8* or negative control were lysed and processed for western blot to detect ZC3H8 and β-TUBULIN protein levels. Densitometry using ImageJ revealed a 25–30% reduction of ZC3H8 levels in cells targeted by shRNA. RT-qPCR was also used to quantify *Zc3h8* levels standardized using *Gapdh*. **b**) Cell lines above were grown in 96-well dishes for 5 days and assayed for cell proliferation using a luciferase viability assay. Significance was calculated using ANOVA at *p* < 0.001. **c**) Cells from above were grown in soft agar overlays for 14 days and imaged using light microscopy. **d**) Average number of soft agar colonies was measured after 14 days using ImageJ. Significance was calculated by Student’s t-test at *p* < 0.01. **e**) To test cell migration, a wound healing assay was done and the width of the gap between cells was measured over time. Significance was calculated by ANOVA at 10 h *p* < 0.05, at 24 h *p* < 0.01. **f**) Transwell invasion assay was done with cV1A 03–31 cells transfected a vector driving expression of shRNA for negative control, *Zc3h8*, or the latter cells additionally transfected with a vector driving expression of human ZC3H8 (not subject to the shRNA-mediated knockdown). Migrated cells were fixed and stained with crystal violet. **g**) Quantification of cells that invaded through the Matrigel transwell assay. Control and rescued cell counts were not significantly different, significance between each and the Zc3h8 siRNA was *p* < 0.01 by Tukey’s post-hoc analysis. **h**) Images of 3-D spheroid cultures of the transfected cells. **i**) Quantification of spheroid growth from H. Statistical difference was calculated by ANOVA at *p* < 0.05. **j**) BALB/c mice were injected subcutaneously with cV1A 03–31 mouse mammary cells transfected with silencing vector targeting *Zc3h8* or negative control. Upon reaching 1 cm diameter or following 8 weeks, tissue was removed and weighed. *P*-value of < 0.01 was determined by Student’s t-test. **k**) Kaplan Meier plot detailing time of appearance of tumors. Experiments for this Figure were performed in triplicate and error bars represent standard deviation. Images are representative of typical results. For all segments, * indicates *p* < 0.05 and ** indicates *p* < 0.01

Since *Zc3h8* expression is elevated in many tumor cell lines, we stably transfected cells with vectors generating shRNA targeting *Zc3h8* mRNA at one of two sites to generate tumor cell lines with reduced expression of *Zc3h8* or negative control shRNA targets. Cell lines with very low levels of *Zc3h8* expression, including CRISPR knockouts from 4 independent mouse mammary cell lines, did not survive in culture for more than a few cell divisions, however, we were able to generate cells with reduced expression that could survive. These cells have a decrease in ZC3H8 of about 25–30% as determined by RT-qPCR, western blot and densitometric analysis using ImageJ (Fig. 2a, Additional file 1: Figure S1A). Mouse mammary tumor cells (cV1A 03–31) that have knocked down expression for *Zc3h8* have a slower proliferation rate than cells transfected with the control pSilencer vector as determined by cell viability assay (Fig. 2b). Strikingly, cells with reduced expression of *Zc3h8* lacked the ability to form colonies in soft agar compared to control (Fig. 2c, d). Control cells transfected with an empty vector readily formed large 3D colonies suspended in semi-solid medium within 2 weeks, but no large colonies were observed in cells knocked down for *Zc3h8*. Only single-cells could be seen in these dishes.

Cell lines with reduced expression of *Zc3h8* also have slower rates of cell migration compared to controls in wound healing assays. Mouse mammary carcinoma cells were tested for their ability to fill in a gap generated for a wound healing assay. Control cell lines migrated into the gap much more quickly than cell lines with reduced *Zc3h8* (Fig. 2e). We also plated these cells in the upper chamber of a transwell invasion assay growth chamber. Control cells readily crossed the extracellular matrix gel and porous membrane to the lower chamber, however, very few cells with reduced expression of *Zc3h8* were able to migrate through the membrane (Fig. 2f). Human ZC3H8 has a very similar amino acid sequence to mouse ZC3H8. Human ZC3H8 expression, however, is not affected by the shRNA designed to target the mouse mRNA. We therefore transfected cells with reduced expression of mouse *Zc3h8* with a vector driving expression of human ZC3H8 and repeated the transwell invasion assay. We found that human ZC3H8 was able to rescue the knockdown phenotype and these cells showed migration efficiency similar to control cells (Fig. 2g). Knockdown of *Zc3h8* expression also led to the cells’ inability to grow as spheroids, whereas the control cells with original *Zc3h8* levels grew rapidly under these conditions (Fig. 2h, i ). We wanted to know if our results from studying *Zc3h8* in cells in culture could translate into changes in tumor growth in vivo. To test this we injected control cells and cells with reduced expression of *Zc3h8* into the mammary glands of syngeneic female BALB/c mice. Tumors were allowed to form in the mice and were removed after 8 weeks or upon reaching 1 cm in diameter. Cells with decreased expression of *Zc3h8* grew more slowly in vivo, and formed significantly fewer and smaller solid tumors than control cells (Fig. 2 j, k). This indicates that while reduced expression of *Zc3h8* leads to a slight decrease in cell proliferation overall, in a more challenging environment cells cannot proliferate.

Measurements of these growth properties were carried out using another cell line (cV1A 01–51) in which *Zc3h8* was targeted using the same shRNA (C) and in the cV1A 03–31 cells using a different siRNA target (A). These experiments showed that cells with reduced ZC3H8 have decreased migration and invasion rates, an inability to form colonies in semi-solid medium and reduced tumor-forming ability (Additional file 1: Fig. S1 B-E). Taken with the overexpression of this gene in tumor cells, *Zc3h8* appears to promote tumorigenesis. *Zc3h8* may also have a role in fundamental cellular functions since we were unable to attain complete knockout of gene expression in cell culture using CRISPR.

### Overexpression of Zc3h8 promotes cell growth and migration in vitro

**Fig. 3:**
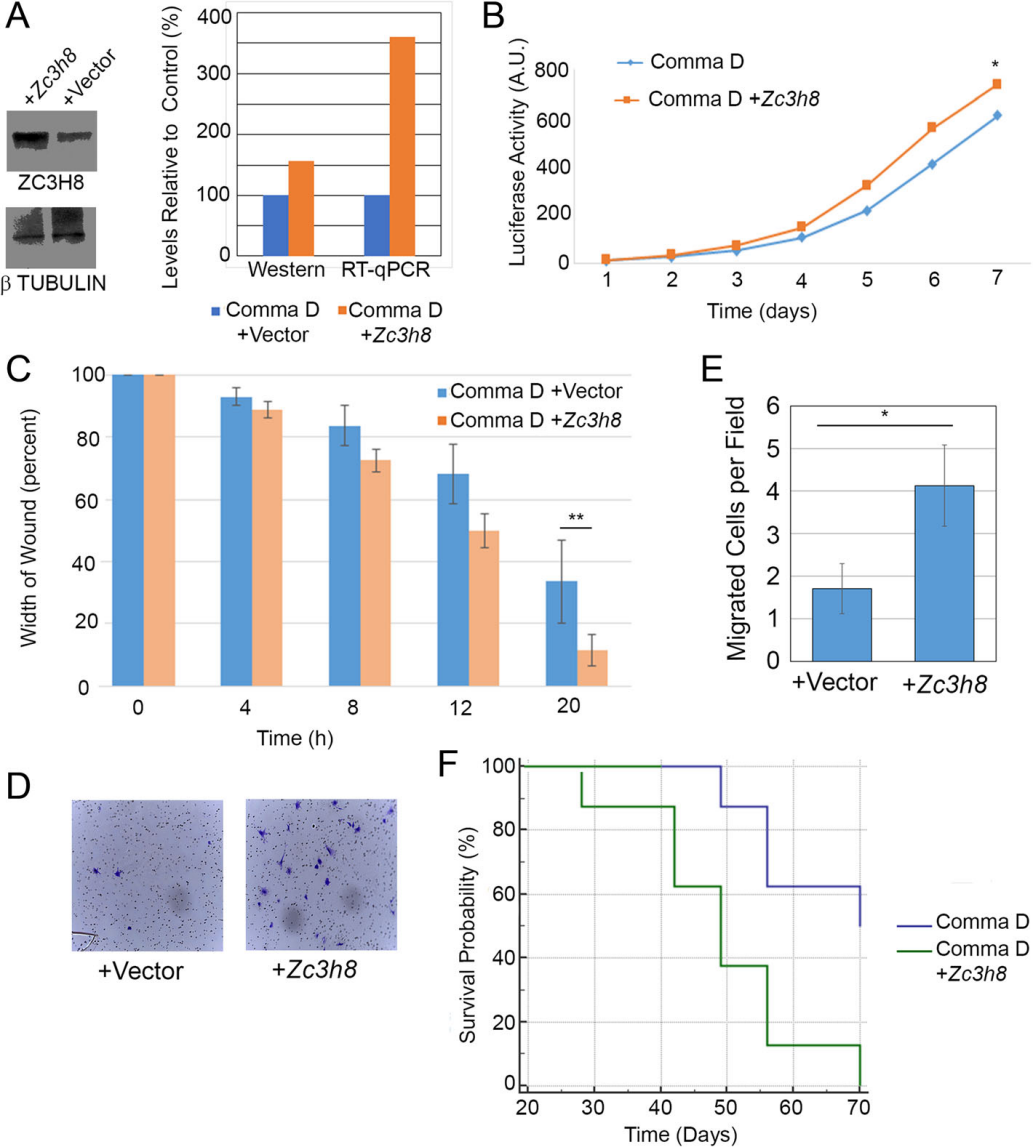
Overexpression of *Zc3h8* in COMMA-D cells leads to increased aggressive behavior. **a**) Mouse mammary COMMA-D cells were stably transfected with empty vector or vector driving *Zc3h8* under control of the EF6 promoter and processed for western blot. Images were quantitated using Image J, and results of RT-qPCR are also shown. **b**) COMMA-D cell lines were grown in 96-well plates and tested for proliferation using luciferase viability assay. ANOVA was used to determine significance *p* < 0.05. **c**) Wound healing assay using stably transfected COMMA-D cell lines. ANOVA was used to assess significance at *p* < 0.01. **d**) and **e**) Transwell invasion assay and quantification with COMMA-D stable cell lines. Experiments were performed in triplicate, significance determined to be *p* < 0.05 by Student’s t-test. For all the above experiments error bars represent standard deviation, and images are representative of typical results (* indicates *p* < 0.05 and ** indicates *p* < 0.01). **f**) BALB/c mice were injected with syngeneic COMMA-D cells that had been transfected with a control vector or one driving expression of ZC3H8 and were monitored for the appearance of tumors for 10 weeks

Decreased expression of *Zc3h8* in carcinoma cells decreased their growth and migration abilities and may have made these cells less aggressive. We wanted to see if non-cancerous cells could gain an aggressive phenotype if *Zc3h8* was overexpressed. To test this we used COMMA-D cells, a mouse mammary epithelial cell line that has normal mammary characteristics [23]. Cells were stably transfected with mouse *Zc3h8* using a strong promoter or empty vector alone and assessed for expression by RT-qPCR and western blot (Fig. 3a). COMMA-D cells with approximately four fold higher expression levels of *Zc3h8* showed faster proliferation in cell viability assays (Fig. 3b). Also, cells with higher levels of ZC3H8 were capable of migrating more quickly as measured by a wound healing assay (Fig. 3c). As expected, control COMMA-D cells were not very capable of migrating through Matrigel in a transwell invasion assay, however, expression of ZC3H8 increased the ability of these cells to migrate (Fig. 3d, e). COMMA-D cells overexpressing *Zc3h8* formed tumors in syngeneic BALB/c mice more rapidly than cells transfected with a control vector (Fig. 3f). The control transfected COMMA-D cells also did form some tumors, consistent with previous results [28]. Overexpression of *Zc3h8* in COMMA-D cells demonstrates that these noncancerous mammary cells acquire some properties of transformed cells – faster proliferation, faster migration, invasion potential and ability to form tumors in vivo. These data complement the experiments done with tumor cells that have reduced *Zc3h8* expression and the opposite phenotype.

A previous study demonstrated that ZC3H8 protein bound to an intronic regulatory region of the *Gata-3* gene in T cells, decreasing *Gata-3* expression and thus playing a major role in T cell development [29]. We hypothesized that ZC3H8 might have a similar role in mammary cells as *Gata-3* is a major determinant of mammary cell fate [30, 31]. Ectopic overexpression of *GATA-3* was shown to lead to reduced tumor outgrowth in the mammary fat pad and loss of metastatic potential of aggressive human tumor cell lines [32]. We assessed the relative levels of expression of *Zc3h8* and *Gata-3* in our panel of cell lines, and in knocked-down and overexpressing derivatives. Tumor cell lines demonstrated varying ratios of *Zc3h8* to *Gata-3* levels, while stable overexpression did not decrease *Gata-3* levels (Additional file 1: Figure S1 F, G).

### Localization of ZC3H8 to nuclear/PML bodies

**Fig. 4:**
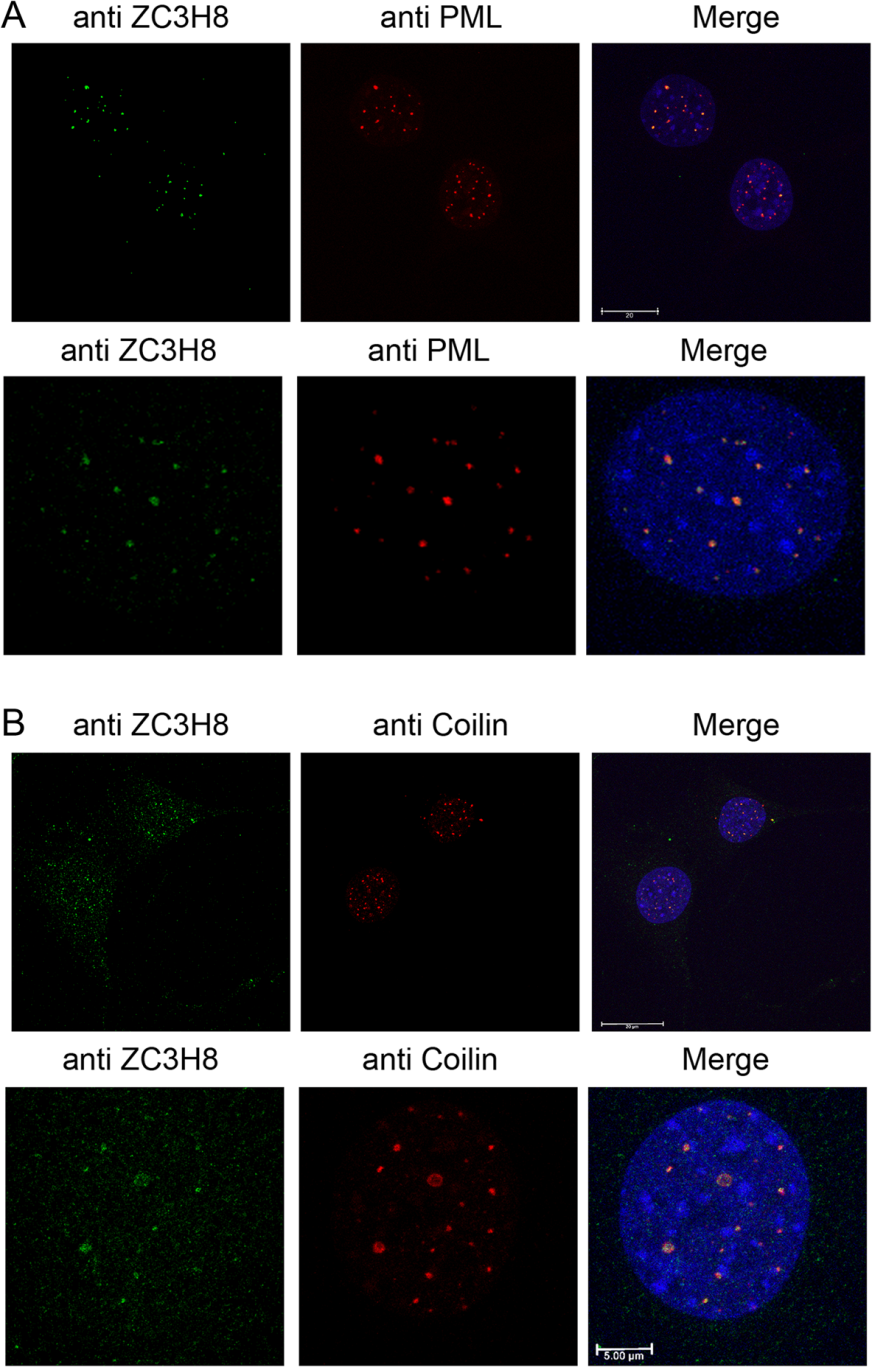
Localization of ZC3H8 to nuclear bodies. **a**) Immunofluorescence microscopy and co-localization of endogenous ZC3H8 and PML to PML bodies in cV1A 03–31 mouse mammary carcinoma cells. **b**) Co-localization of ZC3H8 and COILIN by immunofluorescence microscopy. Images of single nuclei are shown in the lower panels and depict z-stack images. In low magnification images, scale bars represent 20 μm, and represent 5 μm in high magnification images

To elucidate the function of ZC3H8, we determined the subcellular localization of the endogenous protein using immunofluorescence confocal microscopy. In cells derived from tumors or from the normal mammary gland, ZC3H8 is located in numerous foci within the nucleoplasm (Fig. 4a and Additional file 2: Figure S2A, B). These foci resemble known nuclear domains, specifically PML bodies, Cajal Bodies, or nuclear paraspeckles. A previous study using microscopy screens to identify novel components of nuclear bodies found that ZC3H8 colocalized with p54, an essential component of nuclear paraspeckles that retain A to I –edited mRNAs [17]. However, ZC3H8 and other LEC components have been found in Cajal bodies, sites of processing and assembly of snRNAs and snRNPs [14, 15]. PML bodies contain a variety of proteins involved in genome maintenance and cellular defense responses [33].

We tested localization of ZC3H8 with PML as a marker for PML bodies, COILIN as a marker for Cajal Bodies, and NONO as a marker for paraspeckles. ZC3H8 has strong co-localization with PML and partial co-localization with COILIN (Fig. 4a, b). The localization of PML, COILIN, CK2, and ICE2 was confirmed in an additional mouse cell line, COMMA-D (Additional file 2: Figure S2A). SMN, another Cajal body marker, colocalizes with COILIN and ZC3H8 (Additional file 2: Figure S2B). ZC3H8 does not appear to co-localize with NONO (data not shown). Cajal body COILIN and additional PML body marker DAXX colocalized in HeLa cells (Additional file 2: Figure S2C), although lack of cross-species reactivity of antibodies detecting DAAX and ZC3H8 prevented us from linking all three markers in either species. Cells from lines with decreased ZC3H8 showed slightly increased numbers of PML bodies (Additional file 2: Figure S2D). These data indicate that ZC3H8 may be located in either PML bodies, Cajal Bodies or both. PML bodies participate in a surprising variety of functions, including regulation of transcription, apoptosis, DNA repair, telomere maintenance, and antiviral defense [34], while Cajal bodies are largely focused on RNA processing. As ZC3H8 contains potential nucleic acid binding motifs, its predicted structure supports these localization data.

### Disruption of ZC3H8 and PML bodies with casein kinase 2 inhibitor

**Fig. 5:**
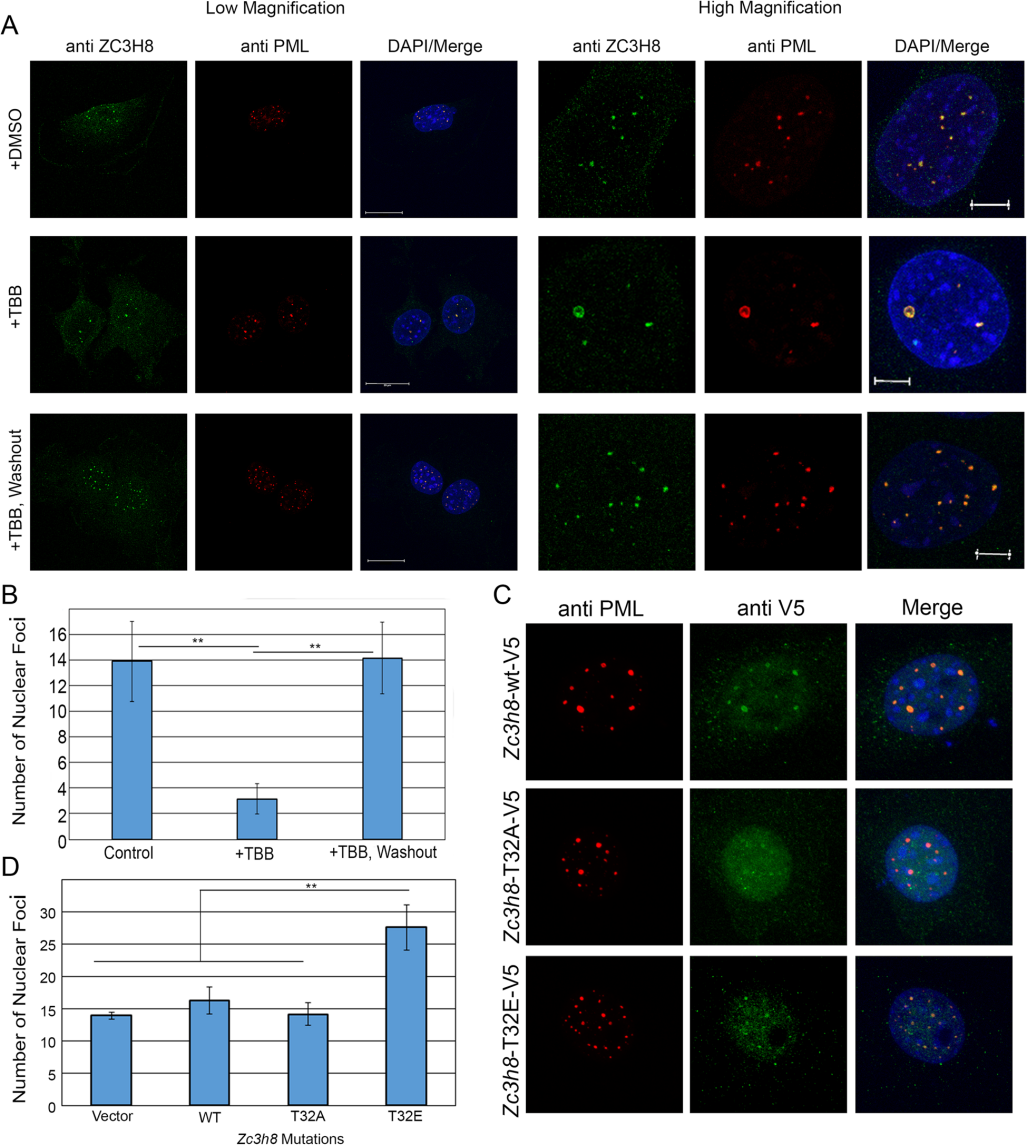
ZC3H8 and PML localization is altered by CK2 inhibitor TBB. **a**) cV1A 03–31 cells were treated with 10 μM of the CK2 inhibitor TBB for 2 h and processed for immunofluorescence using antibodies against PML and ZC3H8. Control cells were treated with DMSO only, and washout cells had the TBB media replaced with fresh media for 2 h. **b**) Quantification of the number of nuclear foci stained with PML and ZC3H8 (PML Bodies) treated with DMSO, TBB, or TBB and washout. Differences between control and TBB washout were not significant, but both were significantly different from TBB treated cells, *p* < 0.01. **c**) Cells transiently transfected with vector encoding ZC3H8-WT, ZC3H8-T32A, or ZC3H8-T32E phosphorylation mutants and processed for immunofluorescence against PML (red) and V5 tag (green). **d**) Quantification of PML-stained nuclear bodies in cells transfected with *Zc3h8* phosphorylation mutants. Differences between vector, WT, and T32A cells were not significant, but all differed from the T32E mutant. Experiments were performed in triplicate, error bars represent standard deviation, and *p* values were determined by Tukey’s post hoc at *p* < 0.01., Images are representative of typical results. In low magnification images, scale bars represent 20 μm, and represent 5 μm in high magnification images

ZC3H8 contains a consensus sequence for phosphorylation by CK2 at T32 (Fig. 1a). Interestingly, PML is also regulated by CK2 and is degraded as a result of phosphorylation via ubiquitination [35]. We decided to see if the CK2 inhibitor, TBB [36], alters ZC3H8, PML, or COILIN localization to their respective nuclear domains. TBB was most effective when cells were treated with the inhibitor in serum-free media for 2 h with 10 μM of TBB. The CK2 inhibitor had dramatic effects on the localization of both PML and ZC3H8 in treated cells. TBB treatment causes both PML and ZC3H8 to become diffuse in the nucleoplasm and associate with far fewer (albeit larger) nuclear foci (Fig. 5a). COILIN localization was unaffected (Additional file 3: Figure S3A). Additionally, ZC3H8 and PML localization can be restored by washing out TBB and allowing the cells to recover for 1 h (Fig. 5a). The number of nuclear foci containing PML or ZC3H8 were quantified for each treatment with or without TBB (Fig. 5b). An alternative CK2 inhibitor quinalizarin gave identical results (Additional file 3: Figure S3B).

We reasoned that ZC3H8 localization may be dependent either on proper PML localization or direct phosphorylation by CK2. We used site-directed mutagenesis to alter T32 within the phosphorylation site of ZC3H8. cV1A 03–31 cells were transiently transfected with wild type ZC3H8 -V5-His, T32A mutant ZC3H8 -V5-His, or T32E mutant ZC3H8 -V5-His and localized with anti-V5 immunofluorescence so that only the vector-encoded ZC3H8 product was visualized. Wild type ZC3H8 overexpression leads to the appearance of larger nuclear bodies than those in cells with only endogenous ZC3H8 expression (Fig. 5a, c). When threonine is mutated to alanine, removing the CK2 phosphorylation site, ZC3H8 is localized to PML bodies, and there is little effect on PML localization and body number (Fig. 5c, d). Interestingly, T32E ZC3H8 increases the number of PML bodies in the nucleus and they are smaller than wild type (Fig. 5c, d). Overall, PML protein levels are not changed by the ZC3H8 mutants (Additional file 3: Figure S3C). When glutamic acid with a negative charge is substituted for the wild type threonine, this protein may mimic a constitutively phosphorylated ZC3H8. This observation supports the idea that inhibition of CK2 with TBB prevents phosphorylation of ZC3H8 and alters nuclear structure and PML body number and size independent of PML phosphorylation.

**Fig. 6:**
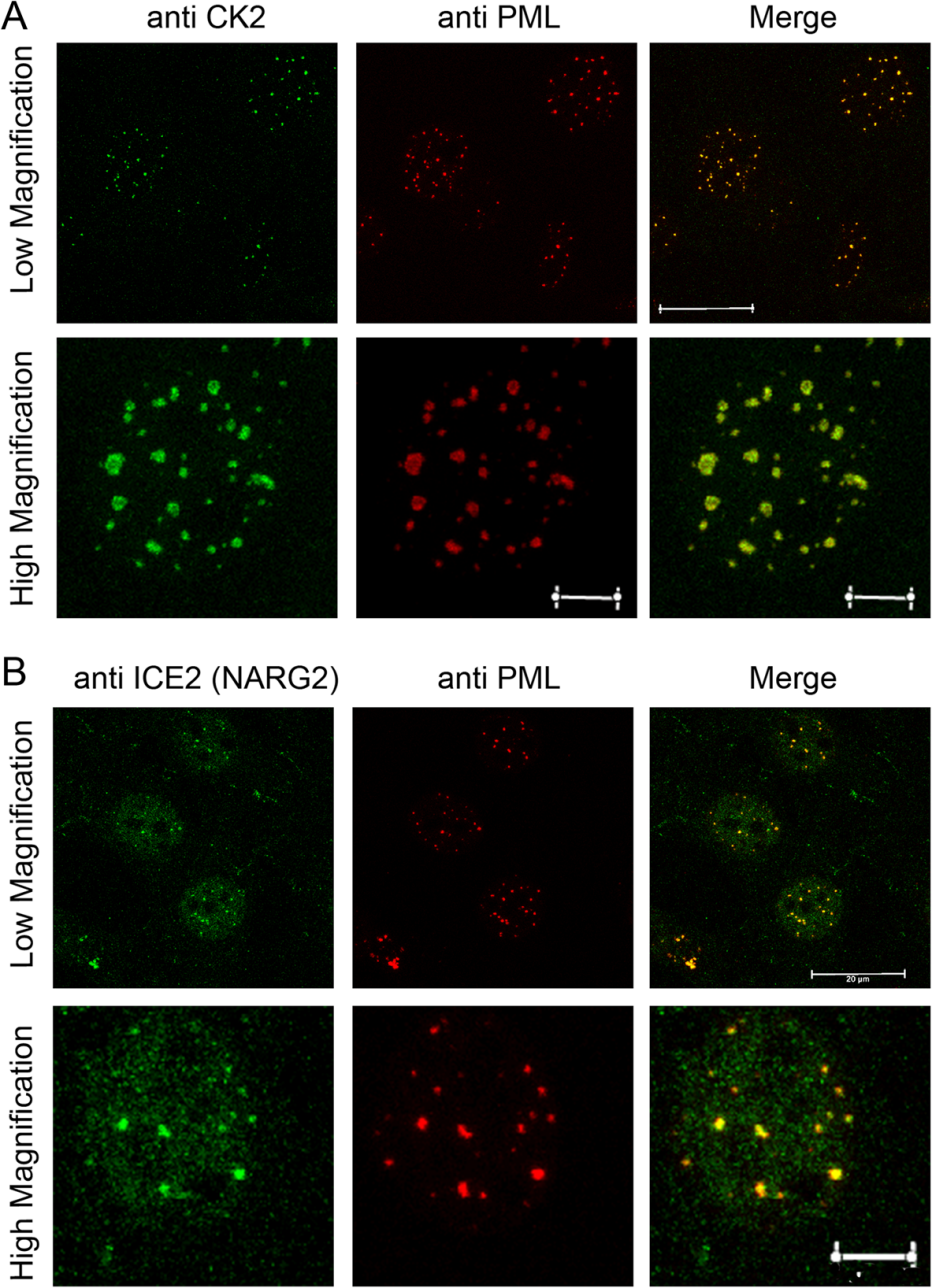
Localization of the Little Elongation Complex and CK2 to ZC3H8/PML Bodies. **a**) LEC component ICE2 (NARG2) co-localized with PML bodies by immunofluorescence microscopy. **b**) CK2 co-localized with PML bodies by immunofluorescence microscopy. In low magnification images, scale bars represent 20 μm, and represent 5 μm in high magnification images

ZC3H8 has also been implicated as a possible accessory protein in the little elongation complex (LEC). We co-localized one component of the complex, ICE2, also known as NARG2, with PML bodies in the nucleus (Fig. 6a). Additionally, we found that CK2 localizes to PML bodies (Fig. 6b). These data support the idea that ZC3H8 is localized to PML bodies in the nucleus by CK2 phosphorylation and works with the LEC at these transcriptionally active sites.

## Discussion

Here we demonstrate that a novel component of nuclear bodies, ZC3H8, has a role in regulating cell behavior and promoting tumor formation. The association of nuclear body proteins and cancer is gaining research focus as we learn more about the function and regulation of these structures. Tumor suppressor genes including PML and p53 localize to nuclear bodies, and disruption of these proteins by degradation or post translational modification promotes carcinogenesis [37]. Both PML and p53 are also targets of phosphorylation by CK2 [38, 39]. Another tumor suppressor, SHIP1, also colocalizes with PML, CK2, and p53 in nuclear bodies [40], suggesting that these structures may represent a nexus for cellular growth control. Both p53 and PML play critical roles in apoptosis, which could influence the survival of cells experiencing *Zc3h8* knockout or high overexpression. Indeed, one of the earliest descriptions of ZC3H8 function noted that T-cell specific overexpression of the gene in transgenic mice led to dramatic thymic cell depletion [41]. The fact that very high or very low levels of *Zc3h8* expression have not been sustainable in our hands also suggests an important role for this protein in cell function. Furthermore, elevated levels of CK2 activity results in polyubiquitylation of PML and proteasome-mediated degradation [35, 39]. Loss of PML leads to increased tumorigenesis in vivo [35]. CK2 is an oncogene that regulates PML and ZC3H8-localized nuclear bodies, and is also a potential therapeutic target in the treatment of cancer [42]. However, our data do not suggest that PML levels increase upon CK2 inhibition, although extended periods of exposure to the inhibitor could show a more substantial effect. Nuclear bodies and their factors clearly have important functions for the cell and cellular transformation.

Although ZC3H8 appears to co-localize with PML at nuclear bodies and with COILIN at Cajal Bodies, these two nuclear domains are considered separate parts of the nucleus [34]. PML and Cajal Bodies may form adjacent to each other, and can vary in number and location between different cell lines, different species, different organs, and as a result of infection or transformation [43]. Sun et al. established that a PML component, PIASy, a SUMO ligase, directly interacts with COILIN to create a link between the two bodies [44]. Our current study shows that PML bodies exhibit altered organization in the nucleus when ZC3H8 phosphorylation is altered, but COILIN-containing Cajal Bodies are not apparently affected by blocking CK2-mediated phosphorylation.

Other genes have recently been identified as encoding proteins that have similar zinc-finger motifs (C3H1) to ZC3H8, although the arrangement of these motifs in the primary sequence often varies [45]. Many of these proteins are thought to be RNA-binding proteins and may be involved in post-transcriptional regulation including alterations to the polyA tail. In *Drosophila*, depletion of ZC3H3 results in mRNA with longer polyA tails and inefficient nuclear export [46]. If *Zc3h8* follows this trend, altering expression and localization of *Zc3h8* can have significant implications on numerous genes’ transcription products and overall expression. This could also account for the many changes in cell behavior that we have shown as a result of *Zc3h8* knockdown or overexpression such as altered migration, invasion, proliferation, and growth in either soft-agar or spheroid plates in vitro and tumor formation in vivo.

Furthermore, association of ZC3H8 with the LEC, but not having a direct effect on snRNA transcription, suggests the function of ZC3H8 in this complex may be post-transcriptional. Hu et al. suggest that LEC component ICE1 acts as a scaffold to recruit ICE2, ELL, ZC3H8, and Pol II to promoters where the complex has functions in both initiation and elongation [14]. In this complex, ZC3H8 may act as an accessory to modify or target transcripts as they are produced, as it is not a key player in recruitment, initiation, or elongation of snRNA transcripts. Alternatively, the LEC may be involved in transcription of other classes of genes, for which ZC3H8 may play a more central role. Recently, p53 was demonstrated to disrupt the formation of the LEC by interaction with the ELL subunit, displacing it from interaction with ICE1 and leading to inhibition of expression of some snRNAs [47]. This interaction was also predicted to displace ZC3H8. Nevertheless, overexpression or knockdown of *Zc3h8* would have profound effects on post-transcriptional modifications. This is a direction for future studies.

We have also shown that expression of *Zc3h8* not only alters cell behavior in vitro, but can also promote tumorigenesis in vivo. Tumor cell lines we tested have elevated levels of *Zc3h8* and data from cBioPortal also shows amplification of ZC3H8 in many types of cancer as well as high levels of expression measured by RNAseq [2, 48]. Additionally, we show here that reducing expression of *Zc3h8* in mammary tumor cell lines injected into mice greatly reduces the number and size of tumors that form.

Although a previous report linked *Zc3h8* to a decrease in *Gata-3* expression in T cells [29], we cannot support *Gata-3* regulation by ZC3H8 in mammary cells and tumors. Such an effect would have provided an attractive explanation for the oncogenic activity of ZC3H8, as *Gata-3* is essential for maintenance of mammary cell differentiation, [30, 31] and GATA-3 serves as a positive prognostic factor in human breast cancer [32, 49–53]. However, in our hands *Gata-3* levels did not increase when ZC3H8 levels were low, and *Gata-3* levels did not decrease when ZC3H8 was overexpressed. We were, however, unable to maintain proliferation of cells with the dramatically increased ZC3H8 levels used transiently in the previous report (data not shown).

## Conclusions

We show that the level of *Zc3h8* expression in murine mammary cells influences aggressive behavior in vitro and tumor formation in vivo. We localized ZC3H8 to PML and Cajal nuclear bodies and demonstrate that the number and size of PML bodies is altered by casein kinase 2 phosphorylation, and the integrity of a phosphorylation site in the ZC3H8 N-terminal domain. An additional component of the Little Elongation Complex also localizes to PML bodies. These results provide a novel link between the LEC, PML bodies, and a protein capable of inducing oncogenic behavior.

## Additional files

Additional file 1: **Figure S1.** Reduced expression of *Zc3h8* in a second cell line and using a second targeting shRNA leads to reduced invasive behavior. A) Western blot demonstrating reduced levels of ZC3H8 protein in cV1A 03–31 cells transfected with a vector driving expression of an shRNA targeting site A. B) cV1A 03–31 cells transfected with a vector driving expression of siRNA targeting *Zc3h8* at site A close a wound more slowly than cells transfected with a negative control vector in vitro. ANOVA revealed a significant difference of *p* < 0.05. C) A second tumor cell line, cV1A 01–51, was transfected with a vector targeting *Zc3h8* at site C or negative control. Cells with reduced *Zc3h8* closed a wound more slowly than negative control cells. ANOVA was used to assess significance at 12 h of *p* < 0.001. D) cV1A 01–51 cells with targeted *Zc3h8* also formed smaller growths in syngeneic BALB/c mice than negative control cells. Student’s t-test was used to determine *p* < 0.001. E) cV1A 01–51 cells with reduced *Zc3h8* did not form colonies in soft agar, while those transfected with a negative control formed large growths after two weeks. F) Tumor cell lines were surveyed by RT-qPCR for relative expression of *Zc3h8* and *Gata-3* compared to levels in virgin mammary gland, shown in a scatter plot. G) Overexpression of *Zc3h8* in COMMA-D cells did not lead to a decrease in *Gata-3* levels as determined by RT-qPCR. Error bars represent standard deviation. (TIF 22335 kb)

Additional file 2: **Figure S2.** Nuclear Protein Marker Localization in mouse and human cell lines. A) Localization of ZC3H8, PML, COILIN, CK2 and ICE2 (NARG2) in nuclear bodies in COMMA-D mouse mammary cells. B) ZC3H8, SMN, and COILIN partially co-localize in cV1A 03–31 cells. C) Localization of COILIN and DAXX in HeLa cells. D) Localization of PML in cells transfected with control or *Zc3h8* shRNA vectors. (TIF 41195 kb)

Additional file 3: **Figure S3.** PML alterations in cV1A 03–31 cells treated with CK2 inhibitors or in cells with mutant ZC3H8. A) Treatment of cells with the CK2 inhibitor TBB has little effect on the localization of COILIN, but leads to mislocalization of ZC3H8 and PML. B) Another CK2 inhibitor quinalizarin also results in fewer PML bodies. C) Expression of T32 mutants does not alter PML protein levels as shown by western blot. (TIF 10719 kb)

## Abbreviations

CK2: Casein kinase 2
ELL: Elongation Factor for RNA polymerase II
ICE1: Interactor of Little Elongation Complex ELL subunit 1
ICE2/NARG2: Interactor of Little Elongation Complex ELL subunit 2/ NMDA receptor- regulated gene 2
LEC: Little elongation complex
MED 26: Mediator complex subunit 26
PML body: Nuclear body containing the promyelocytic leukemia protein
snRNA: Small nuclear RNA
TBB: 4,5,6,7-Tetrabromobenzotriazole
