# Supplementary figures and images for: Regulation of the oncogenic phenotype by the nuclear body protein ZC3H8
Source: BMC Cancer. 2018 Jul 24;18:759. doi: 10.1186/s12885-018-4674-1 (PMC6057032; doi:10.1186/s12885-018-4674-1)

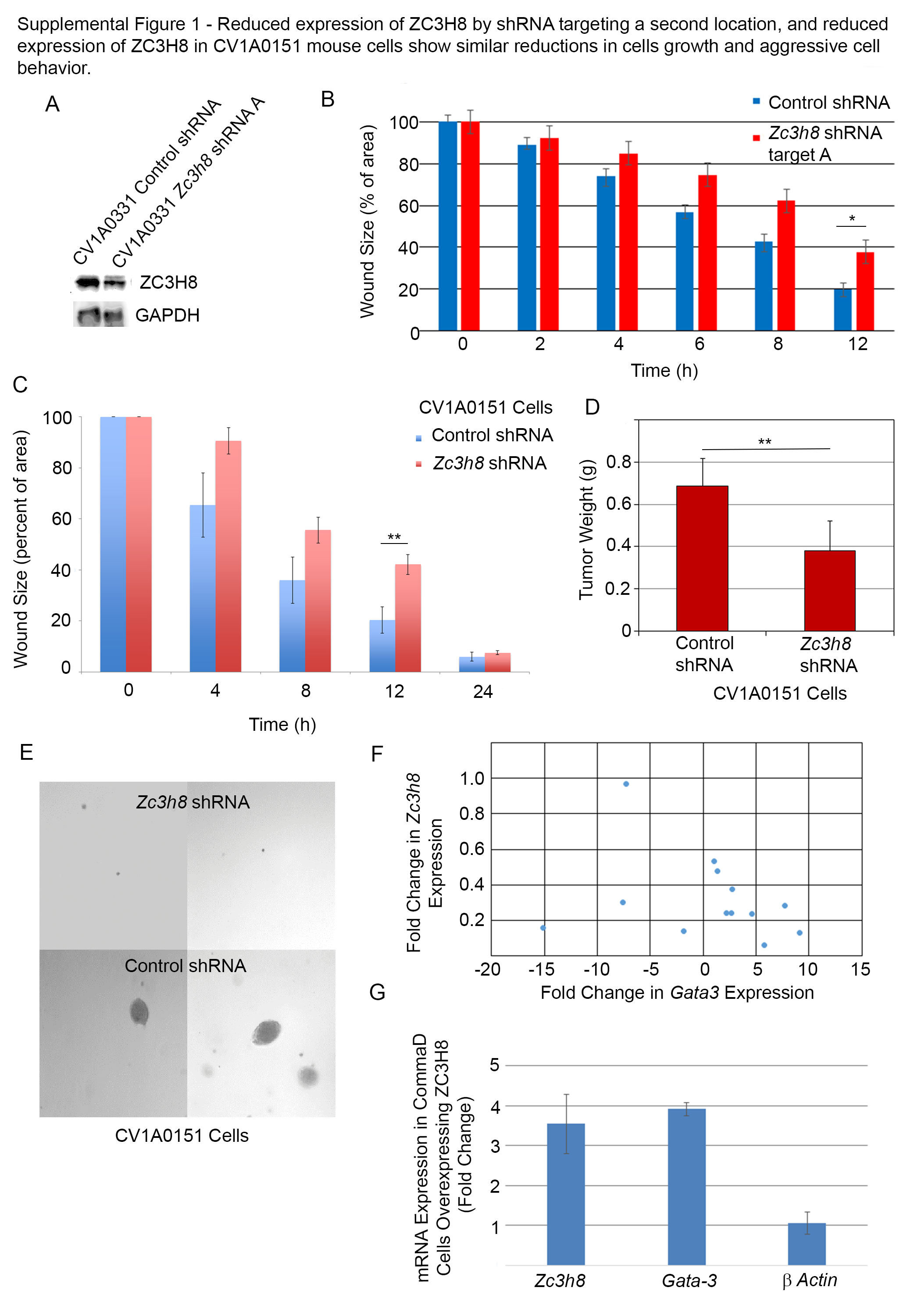

Supplement: Supplementary file 1 — Figure S1. Reduced expression of Zc3h8 in a second cell line and using a second targeting shRNA leads to reduced invasive behavior. A) Western blot demonstrating reduced levels of ZC3H8 protein in cV1A 03–31 cells transfected with a vector driving expression of an shRNA targeting site A. B) cV1A 03–31 cells transfected with a vector driving expression of siRNA targeting Zc3h8 at site A close a wound more slowly than cells transfected with a negative control vector in vitro. ANOVA revealed a significant difference of p < 0.05. C) A second tumor cell line, cV1A 01–51, was transfected with a vector targeting Zc3h8 at site C or negative control. Cells with reduced Zc3h8 closed a wound more slowly than negative control cells. ANOVA was used to assess significance at 12 h of p < 0.001. D) cV1A 01–51 cells with targeted Zc3h8 also formed smaller growths in syngeneic BALB/c mice than negative control cells. Student’s t-test was used to determine p < 0.001. E) cV1A 01–51 cells with reduced Zc3h8 did not form colonies in soft agar, while those transfected with a negative control formed large growths after two weeks. F) Tumor cell lines were surveyed by RT-qPCR for relative expression of Zc3h8 and Gata-3 compared to levels in virgin mammary gland, shown in a scatter plot. G) Overexpression of Zc3h8 in COMMA-D cells did not lead to a decrease in Gata-3 levels as determined by RT-qPCR. Error bars represent standard deviation. (TIF 22335 kb) [file 12885_2018_4674_MOESM1_ESM.tif]

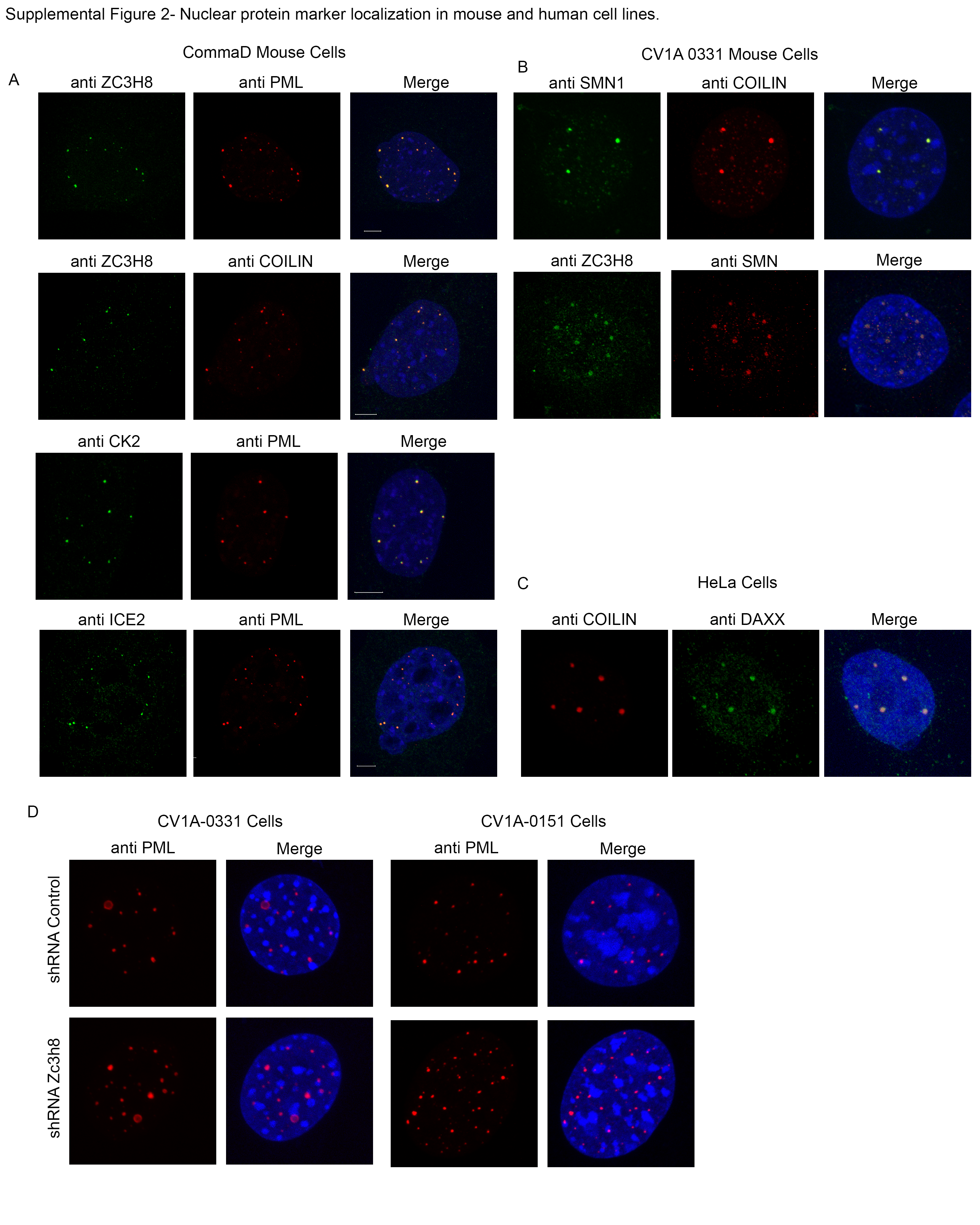

Supplement: Supplementary file 2 — Figure S2. Nuclear Protein Marker Localization in mouse and human cell lines. A) Localization of ZC3H8, PML, COILIN, CK2 and ICE2 (NARG2) in nuclear bodies in COMMA-D mouse mammary cells. B) ZC3H8, SMN, and COILIN partially co-localize in cV1A 03–31 cells. C) Localization of COILIN and DAXX in HeLa cells. D) Localization of PML in cells transfected with control or Zc3h8 shRNA vectors. (TIF 41195 kb) [file 12885_2018_4674_MOESM2_ESM.tif]

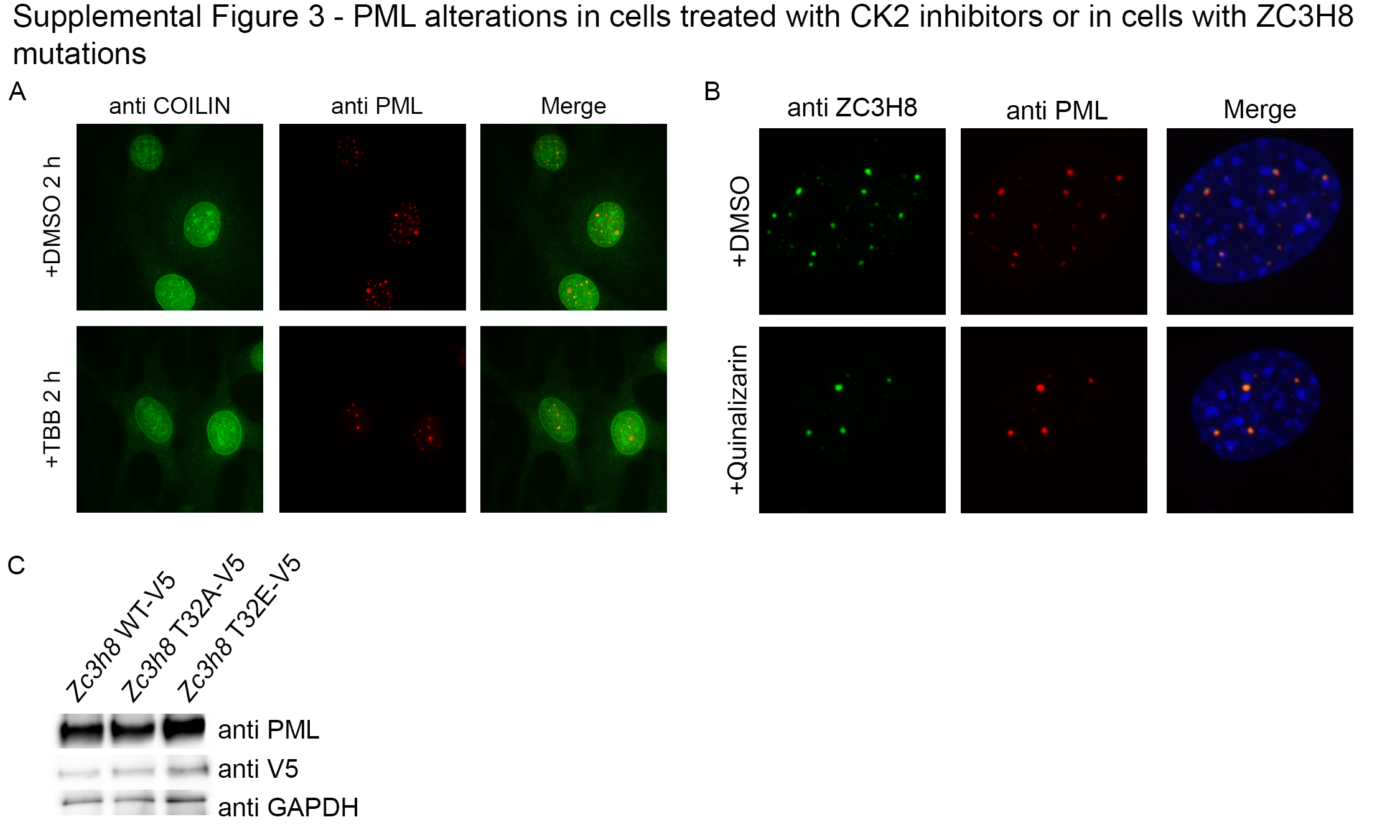

Supplement: Supplementary file 3 — Figure S3. PML alterations in cV1A 03–31 cells treated with CK2 inhibitors or in cells with mutant ZC3H8. A) Treatment of cells with the CK2 inhibitor TBB has little effect on the localization of COILIN, but leads to mislocalization of ZC3H8 and PML. B) Another CK2 inhibitor quinalizarin also results in fewer PML bodies. C) Expression of T32 mutants does not alter PML protein levels as shown by western blot. (TIF 10719 kb) [file 12885_2018_4674_MOESM3_ESM.tif]
